# Alternative Control of *Phragmidium rubi-idaei* Infecting Two *Rubus* Species

**DOI:** 10.3390/plants10071452

**Published:** 2021-07-15

**Authors:** Raluca-Maria Pârlici, Aurel Maxim, Stefania Mirela Mang, Ippolito Camele, Lucia Mihalescu, Vlad Stoian

**Affiliations:** 1 Department of Engineering and Environmental Protection, Faculty of Agriculture, University of Agricultural Sciences and Veterinary Medicine, No. 3-5, Calea Manastur Street, 400372 Cluj-Napoca, Romania; raluca-maria.parlici@usamvcluj.ro; 2 School of Agricultural, Forestry, Food and Environmental Sciences (SAFE), University of Basilicata, Vialedell’Ateneo Lucano 10, 85100 Potenza, Italy; stefania.mang@unibas.it (S.M.M.); ippolito.camele@unibas.it (I.C.); 3 Department of Biology, North University Center of Baia Mare, Technical University of Cluj Napoca, No.76, Victoria Street, 430122 Baia Mare, Romania; lucia.mihalescu@cunbm.utcluj.ro; 4 Department of Microbiology, Faculty of Agriculture, University of Agricultural Sciences and Veterinary Medicine, No. 3-5, Calea Manastur Street, 400372 Cluj-Napoca, Romania; vlad.stoian@usamvcluj.ro

**Keywords:** rust, organic farming, fungistatic effect, conidia germination, blackberry, raspberry

## Abstract

Organic berry plantations have been gaining popularity among farmers during recent years. Even so, farmers experience serious challenges in disease control management, which is a concern in organic farming. *Phragmidium rubi-idaei* (DC) P. Karst is the pathogen responsible for blackberry and raspberry rust disease, one of the most present and active diseases in plantations. The antifungal certified products found on the organic farming market offer the opportunity for an efficient control strategy over plant pathogens in fruit shrub plantations. In this study, 5 natural based products—namely Altosan, Mimox, Canelys, Zitron, and Zeolite—were tested for their fungistatic effect over *P. rubi-idaei.* The experiments were carried out under laboratory conditions, performing observations over the impact of organic products, used at different concentration levels, on rust conidia germination. Moreover, field experiments were conducted in order to evaluate the efficiency of different treatments for rust control on raspberry (‘Polka’, ‘Veten’ and ‘Heritage’) and blackberry (‘Thorn Free’, ‘Chester’ and ‘Loch Ness’) varieties. Data analysis based on ANOVA tests showed significant differences between the tested variants and the control sample at *p* < 0.001. Furthermore, LSD test confirmed differences between all substances tested (*p* < 0.005). The natural products Canelys (formulated with cinnamon) and Zytron (based on citrus extract) have proven the highest inhibitory capacity for conidia germination during in vitro tests registering values of 80.42% and 78.34%, respectively. The same high inhibitory rates against rust pathogen were kept also in the field tests using the same two natural-based products mentioned earlier. In addition, outcomes from this study demonstrated that Zeolite is not recommended for raspberry or blackberry rust control.

## 1. Introduction

*Rubus fructicosus* L. and *Rubus idaeus* L. are among the most popular berries around the globe and are very appreciated for their high vitamin and antioxidant contents. The increasing demand for healthy food and organic products has led to an accelerated growth of organic fruit production in the past 20 years. Blackberry and raspberry are usually cultivated at a small scale and are in a trend of niche production, which makes them very attractive for farmers. In 2000 the world raspberry fruit production was 422,537 tons and by 2019 it had nearly doubled, reaching 822,493 tons, according to FAOSTAT data [1]. Organic farming plays an important role in the world production of berries and the management of pests and diseases is quite a challenge for raspberry and blackberry plantations [2].

Rust is one of the most common diseases within the species of the *Rosaceae* family. Fungi of the genus *Phragmidium* which are responsible for rust disease, often cause galls and lesions on the leaves and young shoots leading to their weakening [3]. On the fruits this pathogen causes uneven ripening. Thus, the losses and the economic impact of this disease in organic plantations set the grounds for concern among farmers [4]. Rust disease not only affects cultural crops like wheat, but also berry plantations that are in a globally challenging growth spurt, mostly in the organic sector. A recent FAO warning expressed great concern regarding the negative impact that diseases have at a global scale, and among them rust was identified as one of the most important around the world in terms of crop losses [1].

Previous studies on natural substances such as essential oils, natural extracts, or biological compounds, which have strong fungicide characteristics, attest to their claims for rust control in organic farming [5,6,7,8,9,10]. Products used in organic farming are based on natural compound formulations, have minimal negative impact on the plant and the environment, are biodegradable, and are formulated on the basis of cost efficiency [11,12,13]. Among the most popular products with antifungal effects are the ones that hold in their composition Chitosan, a polymer extracted from the shells of marine Crustaceans, and which has already proven its efficiency in several laboratory trials [14,15,16]. Moreover, products that contain natural extracts from the bark of the *Mimosa* tree, cinnamon or citrus-based have recognized antimicrobial effects [17,18,19,20,21,22,23,24]. Zeolite, a volcanic tuff whose soil improvement capacities were made known in an extensive study led by Mumpton [25], has other positive agricultural effects subsequently attested, being involved in intensification of soil microbiology and the implicit increase of soil fertility [25,26,27]. Additionally, Zeolite has diverse agricultural uses, being a good candidate for the potentiation of the effects of fertilizers and applied pesticides [28] and exhibiting antimicrobial and fungicidal properties after enrichment with Ag+, Zn+, and Cu+ ions [29,30,31,32].

The optimum soil and plant health, as well as the increased level of biodiversity of the habitat in which organic plantations are placed, are imperative in order to achieve a strategy for effective disease control in the medium and long term. Organic farming is geared towards maintaining the ecosystem balance by applying those methods and products that show respect for nature [33]. In this agricultural system only the use of products and practices recommended by the competent bodies are allowed.

Most of the recent studies on pest control present data concerning the plant pathogen management of bacteria and fungi that generate fruit decay and threaten shelf life [9,32]. The lack of recent data on a plant pathogen like rust fungus, that has such a strong impact on blackberry plantations, weakens the plants and decreases fruiting capacity, led us to undertake a study on how to control it using organic products. Despite the fact that raspberry and blackberry plantations are among the most popular small fruit farms that generate significant profit, there is little recent information in literature regarding scientific studies on the presence of this pathogen on organic berry farms and also concerning its impact on the overall well-being of the affected plants. The background of our research was determined by the next hypotheses: (i) could natural-based products available on the Romanian market control *P*. *rubi-idaei* in organic blackberry and raspberry plantations? (ii) is the applied dose (based on producer recommendations) enough to control the pathogen or there is a need to adapt it to the cultivated blackberry and raspberry varieties? (iii) is there a correlation between the natural-based products effects and blackberry and raspberry varieties? Based on the above presented hypotheses, the aims of the present study were: (1) to investigate the in vitro efficiency of five natural-based products over the germination of rust spores; (2) to test in field the effectiveness of the same five natural-based products to control rust disease in six blackberry and raspberry varieties; (3) to discover the existence of possible correlations between tested rust controlling products and blackberry and raspberry varieties; (4) to evaluate the influence of organic products treatments on the frequency and the severity of the rust disease in both blackberry and raspberry.

## 2. Results

### 2.1. Effect of Organic Products on Rust Conidia Germination

Data analysis revealed significant differences at *p* < 0.001 in the two-way ANOVA and post-hoc LSD (*p* < 0.05) tests for both field and laboratory experiments. For all tested products, excluding the Zeolite which was applied at a standard concentration of 0.5%, reactions to minimum and maximum concentrations recommended by the manufacturer were observed.

The observations for in vitro experiment were made at the first-time interval, 24 h, showing average values close to that of the untreated witness (12) only in the variant Altosan 0.25% (12.67). Low mean values were observed for samples treated with either Zytron 0.15%, Zeolite 0.5%, or Canelys 0.3% (Table 1). Regardless of the different concentrations (0.2% and 0.3%) used for the Canelys product, a lack of activity in the conidia germination level was observed since mean values registered were 0. Observations made at 48 h revealed germination of rust conidia in all tested samples. In particular, low mean values were maintained for Canelys 0.2% (5.0), Canelys 0.3% (6.33), and Zytron 0.15% (7.0). Instead, the Zeolite variant 0.5%, which in the first part of the experiments registered a small average value, compared to the witness, at 48 h showed the highest average value and implicitly was the closest one to the witness. The LSD test, applied to determine the differences between the tested variants, showed that after 24-h from the application of the treatment, no significant differences were found for the Altosan variant 0.5% and the control one. The observations made at 48 h revealed that Altosan 0.5% and Mimox 0.2% did not show significant differences from the other samples analyzed. For all other substances tested, significant differences according to the post-hoc test (*p* < 0.05) were detected.

### 2.2. Field Testing of Organic Products for Blackberry Varieties

The same concentrations, previously tested in the laboratory, were applied through spraying in fields to the batches of ‘Chester’, ‘Thorn Free’, and ‘Loch Ness’ blackberry varieties in both 2018 and 2019. The field experiments were held in the center of Romania, in Transylvania region, and the blackberry culture regime complied with all the rigor requirements of organic agriculture. The analysis of the collected data showed that there were significant differences between the varieties and the treatment variants for each year (Table 2). However, low or no statistical significance resided in the correlation year-treatment and *p*-values were higher than the significance level. Neither the frequency, nor the degree of attack of the rust pathogen, seemed to be influenced by the relation year-variety-treatment. The climate conditions of the two years investigated were uniform for every tested batch. The intensity of the disease was influenced by the interaction between the different treatment variants and the two years observed, as well as the variety tested. The macro short analysis highlighted that the substances tested had a different response in every variety, suggesting a genetic disease resistance for some of them.

From the ANOVA analysis of all three tested blackberry varieties in relations with the two-year period, no impact on the reactions of the plants was found for ‘Loch Ness’ and ‘Chester’. By contrast, ‘Thorn Free’ was influenced by the two different years (Table 3). The treatments applied were also influenced by the blackberry type, *p* < 0.001. Thus, the most sensitive blackberry variety to weather conditions was ‘Thorn Free’. Further studies on the impact of climate and temperature can determine the exact impact on the different reactions to applied products.

The values observed were relatively uniform (Table 4). However, differences from the average of the untreated witness compared to the ones of the tested products, suggested different levels of effectiveness of the substances applied in the control of blackberry rust.

The statistical significance of the results collected from the field batch cultivated with ‘Chester’ blackberry variety was further analyzed by applying the LSD test for significance to a number of three repetitions for each treatment performed (Table 4). Important values for all three parameters (frequency-F, intensity-I, degree of disease attack-DA) were obtained in the case of the control batch. The following high levels of rust attack were observed in the variants treated with Zeolite 0.5% (46.97% DA), which had registered the closest attack level compared to the witness (48.25% DA), and Mimox 0.2% (44.23% DA). Furthermore, the Mimox 0.2% treated batch expressed higher disease attack intensity than the untreated one (71.35%). The disease was frequent on almost half of all the blackberry plants involved in the experiments but the severity was controlled by the applied sprayings. At the opposite pole, the lowest degree of disease attack value was observed for the Canelys 0.3% variant when applied on ‘Chester’ variety (15.27% DA).

Results of the LSD test confirmed the existence of significant statistical differences between the products tested for ‘Thorn Free’ blackberry variety for the two-year period investigated (Table 5). Furthermore, ‘Thorn Free’ variety had the lowest degree of attack in the control batch compared with the other two varieties. Moreover, the intensity and the frequency of rust did not prevail at such a high rate in any of the variants as in ‘Chester’ and ‘Loch Ness’ varieties. The highest levels of disease frequency were found after the application of Zeolite 0.5% and Mimox 0.2%, registering values of 44.83% and 45.50%, respectively. The lowest intensity of rust was recorded for the variants treated with all Canelys and Zytron at different concentrations (Canelys 0.3–25.08%, Zytron 0.15–25.73%, Zytron 0.125–29.42%, Canelys 0.2–29.78%). In addition, the least affected by rust were the plants treated with Canelys 0.3% showing a degree of disease attack of only 7.18%, which was the smallest value observed during the experiments. By contrast to ‘Chester’ and ‘Loch Ness’ varieties, ‘Thorn Free’ expressed a better response to the rust disease most likely indicating a better immune system and moreover a genetic resistance to the disease. For the whole period of study (2018–2019) ‘Thorn Free’ plants were the least affected by rust registering the lowest values for frequency, intensity, and degree of disease attack, respectively. Although the pathogenic fungus was frequent on almost half of the batch the degree of attack was as low as half in severity compared to that recorded on ‘Chester’ and ‘Loch Ness’ varieties.

Among all plants investigated ‘Loch Ness’ blackberry variety had the highest rate of rust infection as demonstrated by data collected from this study. The untreated batch was severely affected by rust with an intensity rate as high as 72.70%. Furthermore, Zeolite 0.5% confirmed its inefficiency to control this pathogen since more than half of the plants were attacked by rust and the intensity level surpassed the one registered in the control variant (75.75%). Variants that do not present significant differences for frequency of the disease were observed in the groups treated with Altosan 0.5% and Mimox 0.2% natural-based products (Table 6). It is worth noting that the variants treated with Canelys 0.3% showed important statistical differences for intensity and degree of disease attack and also registered the smallest values of the average compared to the control. This product distinguished itself in a positive manner towards the other ones tested in this study.

**Figure 1:**
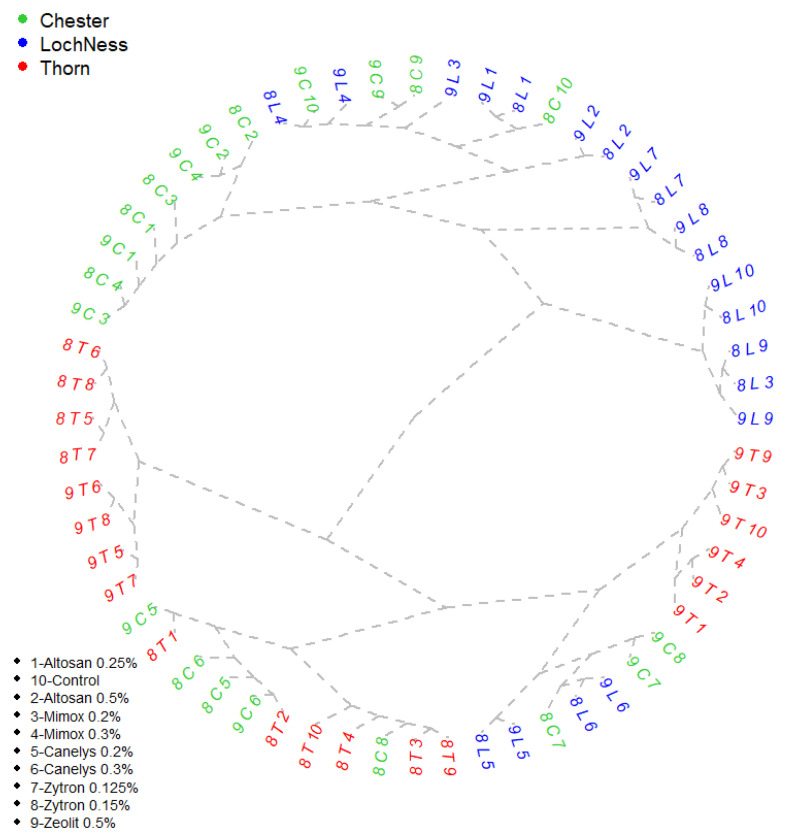
Cluster analysis of field treatments for organic blackberry rust disease control. First number represents the year: 8-2018; 9-2019 followed by the first letter taken from the variety’s name C-’Chester’; L-’Loch Ness’; T-’Thorn’. The second number represents the variant of treatment: V1-Altosan 0.25%; V2-Altosan 0.5%; V3-Mimox 0.2%; V4-Mimox 0.3%; V5-Canelys 0.2%; V6-Canelys 0.3%; V7-Zytron 0.125%; V8-Zytron 0.15%; 9-Zeolite 0.5%; V10-Control. A full description of treatment recipes is provided in Table 12.

For an accurate view of the predictability of treatment results over time, the data collected between 2018–2019, in the field experiments, were organized through a Cluster analysis (Figure 1). In this study, groups within the experiment were generated based on similar treatment response to one or more parameters. Although clusters are used mostly in economy for business predictions, they can be a useful tool to select the products that can create a pattern for rust disease control of organic blackberry.

Variants treated with Altosan 0.5% and Mimox 0.2% were stable and offered for every year investigated similar values for disease frequency (Table 4). Other stable variants were Canelys 0.3% and Zeolite 0.5% as both showed similar differences for frequency and intensity of rust attack. Unstable variants that showed annual uneven parametric differences were: untreated control, Canelys 0.2%, Zytron 0.125%, and Zytron 0.15%. The DA results from 2018 for batches treated with Canelys 0.2% and Canelys 0.3% were very similar with the DA results for Canelys 0.3% variant from 2019, close to 15%. In this case disease control efficiency was 66–68%. Furthermore in 2019, rust was controlled in a very similar manner by Zytron treatments for both tested concentrations (Zytron 0.125%, Zytron 0.15%). Additionally, Zytron was one of the natural-based products that showed little differences in all parameters in both tested concentrations in 2019 but this pattern was not the same for 2018 tests.

‘Thorn Free’ blackberry variety differentiated less stable treatment efficiency for treated variants Altosan 0.25% and Mimox 0.2% in 2018 (Figure 1). The rust attack severity (DA) for variants previously mentioned was 20.38% and 21.55%, respectively. Less efficient variants regarding the rust disease presence were Mimox 0.3% and Altosan 0.5%. They showed very similar values of disease frequency, close to 41% (Table 4). Interestingly, in 2019, the variants: untreated control, Zeolite 0.5%, Mimox 0.2%, Mimox 0.3%, Altosan 0.25%, and Altosan 0.5% were grouped as the least efficient for rust control, showing a uniform disease intensity (50.37–45.80%). Zytron 0.15%, and Canelys 0.3% were the natural products that distinguished themselves by a better disease efficiency (57–71%) for both years analyzed providing stable variants.

The largest cluster was represented by five stable variants for ‘Loch Ness’ variety: Altosan 0.5%, Zytron 0.125%, Zytron 0.15%, Zeolite 0.5%, and control suggesting data uniformity. Altosan 0.25%, Canelys 0.2%, and Canelys 0.3% were also stable variants, although they were situated at different poles of efficiency (Figure 1). All stable variants offered predictable outcomes for each product application. Mimox in both variants (Mimox 0.2% and Mimox 0.3%) was the product which showed low stability, its values of field disease severity registered in ‘Loch Ness’ variety does not qualify it for rust control (Table 6). Altosan 0.25% in both years and Mimox 0.3% for 2019, showed close F values (V1-69.67%; V2-70.67%; V4-69%).

**Figure 2:**
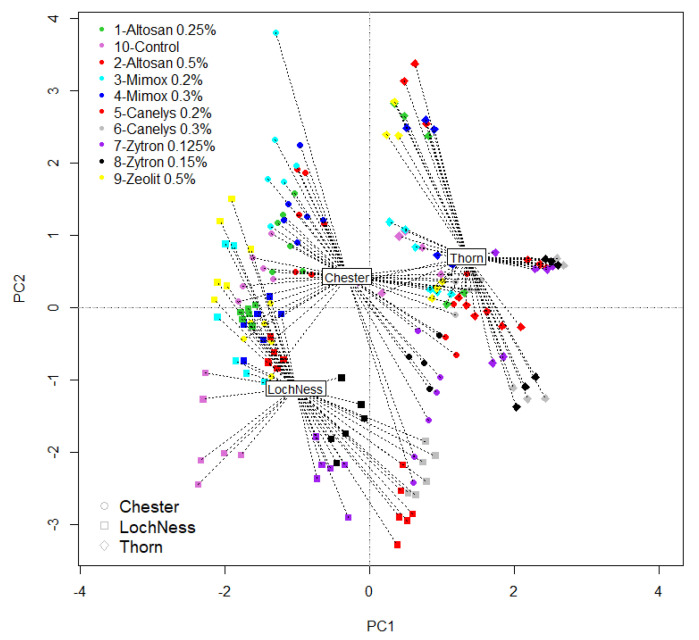
PCA treatment overview in organic blackberry rust disease control. Variants tested: V1-Altosan 0.25%; V2-Altosan 0.5%; V3-Mimox 0.2%; V4-Mimox 0.3%; V5-Canelys 0.2%; V6-Canelys 0.3%; V7-Zytron 0.125%; V8-Zytron 0.15%; 9-Zeolite 0.5%; V10-Control. A full description of treatment recipes is provided in Table 12.

PCA test of data dispersion according to different variables managed to portray a dynamic image of the applied treatments. Thus, data organization between the two axes categorized treatments into mainly positive or negative according to rust disease response to the treatment (PC1-97.39% variance had a positive impact, PC2-2.20% variance had negative impact). ‘Loch Ness’ and ‘Chester’ blackberry varieties have shown similar patterns of data distribution with an important difference: ‘Loch Ness’ variety was more likely to present negative response to some treatments, rather than ‘Chester’ variety (Figure 2). Another important observation to make is that the least stable variants observed were: Zytron 0.125%, Zeolite 0.5%, and Mimox 0.2%. All variants have shown greater dispersion distance than Canelys 0.3% that was the only variant that was on the positive side of the axis for all three blackberry varieties investigated. Although Zytron 0.15% had a positive impact on rust pathogen for all tested variants, ‘Loch Ness’ was the variety that had shown low coherence in response due to climate impact or some specific sensitivity to the disease for this variety. Altosan 0.25% was the only borderline (PC2 point proximity) product for ‘Loch Ness’ and ‘Thorn Free’, its tight point presence on the graph revealed stability only for ‘Loch Ness’. In addition, ‘Thorn Free’ variety had also the most symmetrical and grouped point distribution being the most stable variety in this experiment for rust disease treatment reaction.

### 2.3. In Field Tests of Raspberry Varieties Reaction to Rust Disease Control through Organic Products

During 2018–2019 organic control disease products were tested in an experimental field in Transylvania, in three (‘Heritage’, ‘Veten’, ‘Polka’) raspberry varieties. Data collected from this experiment was analyzed applying two-way ANOVA test for differences, LSD test for significance, and Cluster and PCA synthesis statistics.

The ANOVA test for differences highlighted that the frequency, intensity, and the degree of attack of rust was influenced by each variety and treatment tested for every year of experiment (Table 7). For none of the three raspberry varieties did the year influence the collected data regarding the presence of the pathogen. Besides, the nine treatment variants results did not show differences due to the influence of the year. Likewise, the accumulation of factors: year, variety, variant, did not produce differences in the collected data as far as the attack of *P*. *rubi-idaei* was concerned. On the other hand, the data collected was affected by the interaction between the different applied treatments and the raspberry varieties.

From the analysis of differences for each tested raspberry variety as far as the year’s influence on the collected field information is concerned, ‘Polka’ was the only one affected in each disease aspect (frequency, intensity, degree of disease attack). The frequency and intensity of the rust disease attack, in ‘Heritage’, respectively ‘Veten’ varieties, were not reached through the annual climate impact. Differences between the two years investigated for the severity of the disease attack were found both for ‘Polka’ and ‘Veten’ varieties, although the latter had a *p. val* ≤ 0.001. All tested products had shown a different response for every variety analyzed and the influence of this factor was obvious (Table 8).

Differences regarding the disease presence for every tested product were analyzed for significance for the whole-time frame of the organic raspberry rust disease control experiment. The LSD test highlighted that statistical significance for differences was evident in case of Mimox 0.2%, Mimox 0.3%, and the control batch for ‘Heritage’, control for ‘Polka’, Canelys 0.3%, and control for ‘Veten’ variety (Table 9). Furthermore, the control batch presented the highest frequency rate of the disease for both ‘Polka’ and ‘Veten’ varieties. Zeolite 0.5% offered high values, 39% for ‘Polka’, and 40.50% for ‘Veten’ varieties. The data collected from the ‘Heritage’ variety batch differed from the other varieties analyzed. Frequency of the rust disease in the control was lower than one registered for Mimox 0.2% (52.33% F) treated variant. Zeolite had a lower disease frequency than *Mimosa* formulated product (47.33%) for ‘Heritage’ variety. The lowest values for disease frequency compared to the untreated variant unanimously reported for Canelys 0.3%, Zytron 0.15%, and Canelys 0.2% natural-based products had important impact on the rust disease frequency in all three raspberry varieties investigated. For ‘Polka’ and ‘Veten’ varieties the response to treatment was similar and Canelys 0.2% had the second-best outcome and Zytron 0.15% next. On the other hand, Zytron 0.15% registered the lowest disease frequency value, followed by Canelys 0.2%.

Raspberry varieties investigated exhibited important disease intensity values for Zeolite variant, 63.03% (‘Heritage’), 45.48% (‘Polka’), 51.95% (‘Veten’). Likewise, the untreated variant reported higher values (64.1%). ‘Heritage’ variety also revealed a high disease intensity for Altosan 0.25% and Mimox 0.2% variants in this exact order (Table 10). This differed from ‘Polka’ and ‘Veten’ varieties where Mimox in both treatment variants, V3 and V4, was the third in importance compared to the control while the Altosan 0.25% came afterwards. The lowest intensity of the disease attack was noted for plants treated with Canelys 0.3% in all raspberry varieties. Next lowest values were registered for Zytron 0.15%, 31.42% (‘Heritage’), and 28.47% (‘Polka’), respectively. Canelys 0.2% expressed the second lowest value for ‘Veten’ raspberry variety, Zytron 0.15% (V8) followed in ranking. All means from the tables where intensity, frequency, and degree of disease attack are shown, sharing a common letter do not present statistical significant differences.

The severity of the rust disease presence in the raspberry plantation was appreciated for two years in a row, 2018–2019. Furthermore, the reaction of each tested product was sought for all varieties highlighting the variants that show significance. Thus, for ‘Heritage’ variety the variants bearing significant differences for the whole experiment were: untreated witness, Zeolite 0.5%, Mimox 0.2%, and Mimox 0.3%. ‘Polka’ variety presented the least significant differences for all tested products (Table 11). ‘Veten’ raspberry had the highest difference only for Canelys 0.3% treated batch. The smallest degree of disease attack values was reported for Canelys 0.3% treatment variant in all raspberry varieties investigated. Canelys 0.2% showed a decreased rust attack severity, second in ranking for ‘Veten’ variety. Canelys 0.2% was not as important in treating rust for ‘Polka’ and ‘Heritage’ varieties, instead, for these two, Zytron 0.15% was more efficient. The least efficient, as usual, was Zeolite 0.5%. Mimox 0.2% and 0.3% also proved a decreased efficiency for all varieties analyzed in both variants applied. Altosan 0.25% had little impact on rust attack degree for the observed varieties. Zytron 0.15% managed to control the rust pathogen in a percentage more or less close to median. From the analysis of all data collected there were significant differences for some of the tested products for each raspberry variety analyzed, suggesting an influence over this outcome from the climate, the genetic resistance, the ferocity of the disease, or a combination of these factors.

**Figure 3:**
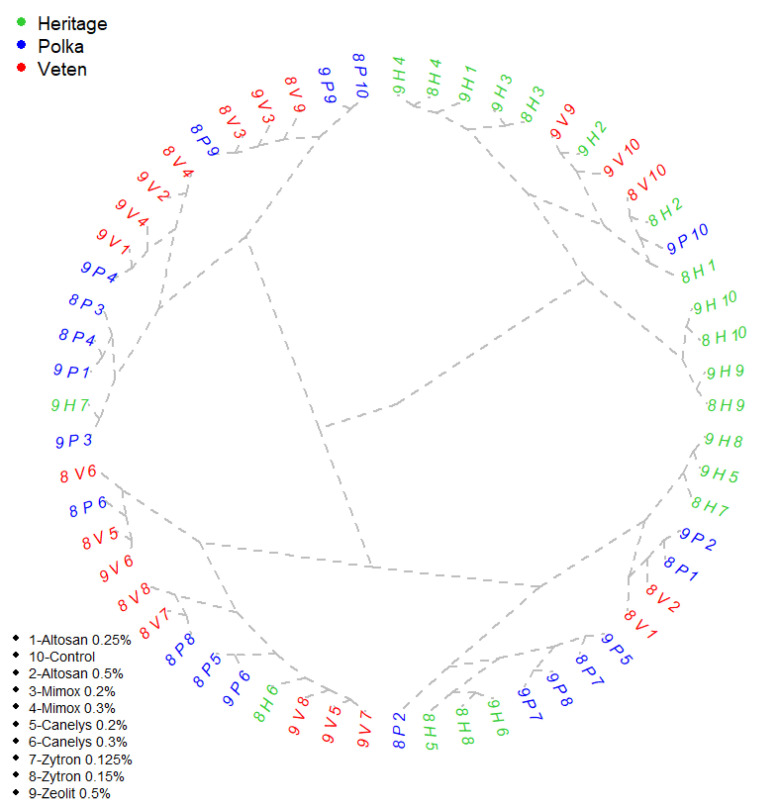
Cluster analysis of field treatments for organic raspberry rust disease control. First number represents the year: 8-2018; 9-2019, followed by the first letter taken from the variety’s name H-’Heritage’; P-’Polka’; V-’Veten’. The second number represents the variant of treatment: V1-Altosan 0.25%; V2-Altosan 0.5%; V3-Mimox 0.2%; V4-Mimox 0.3%; V5-Canelys 0.2%; V6-Canelys 0.3%; V7-Zytron 0.125%; V8-Zytron 0.15%; 9-Zeolite 0.5%; V10-Control. A full description of treatment recipes is provided in Table 12.

Raspberry field data collected was organized in cluster groups to evaluate variants behavior for each variety. Usually, groups were formed around a common value for a parameter creating a replicable response pattern. Most of the tested variants showed stability over time but there were annual differences for some varieties. Altosan was the product that bared the most frequent instability in all varieties investigated (Figure 3). Altosan 0.5% was unstable for ‘Polka’ and ‘Veten’ varieties for both years and formed small clusters with Altosan 0.25% with similar degree of disease attack values, 12.27% for Altosan 0.25% in ‘Polka’ and 12.62% for Altosan 0.5% in ‘Veten’ varieties (Table 11). Furthermore, for ‘Polka’ and ‘Veten’ varieties, Altosan 0.5% performed close values for disease frequency of 30% and 30.33%, respectively. This variant was also unstable for ‘Heritage’ variety. The same product applied in lower concentration (Altosan 0.25%) formed important clusters for different years. Interestingly, Altosan 0.25% performance in 2019 formed a cluster with the one of Mimox 0.2% and Mimox 0.3% during 2018–2019. In rust disease control for ‘Heritage’ variety, the 2018 performance for V1 grouped in a cluster with Canelys 0.2% and Zytron 0.125% in 2018 and Zeolite 0.5% and control—in both years. Similar values for rust disease intensity (29.97–31.4%) and degree of attack (9.07–9.77%) grouped the most efficient treatments in ‘Heritage’ raspberry variety: Canelys 0.3%, Canelys 0.2%, and Zytron 0.15%. By contrast, the least efficient group for pathogenic control of rust in this cluster analysis was Altosan 0.25%-Mimox0.2%-Mimox 0.3%.

Variants that do not present stability for ‘Polka’ variety were: Altosan 0.25%, Altosan 0.5%, Mimox 0.2%, Canelys 0.3%, and the untreated control as they provided different treatment response for every year investigated. At the opposite pole were Mimox 0.3% and Zytron 0.125%, providing a probability trait for treatment response. Rust disease intensity (38.90–42.47%) and frequency (32.33–34.67%) had shown uniform results for both experimental years for Altosan 0.25% and Mimox 0.2–0.3% variants (Table 9 and Table 10). The same parameter uniformity was exercised by Canelys 0.2%, Canelys 0.3%, and Zytron 0.15%, that grouped up towards a disease control efficiency of 60.7–67.3%. Important values in disease management were collected in the first year for Canelys 0.2% and Zytron 0.15% group around Zytron 0.125% median performance for the two-years of the experiment.

The cluster analysis outlined a similar group forming pattern for both ‘Polka’ and ‘Veten’ varieties with relatively homogeneous data sets. For these raspberry varieties, the product Altosan delivered similar group connection as far as frequency is concerned, offering similar values (30–32.17%). Control and Mimox (both concentrations) were the only variants that presented stability for ‘Veten’ variety. Canelys 0.3% and Zeolite 0.5% presented opposite degrees of disease attack outcomes in rust control and had shown the lowest stability. All other variants grouped according to similar disease reaction to treatments for different years but did not present stability although they formed groups. For ‘Veten’ variety in 2018 various variants offered a similar efficiency in rust disease control (Canelys 0.2%, Zytron 0.125% and Zytron 0.15%). In the following year similar occurrence of the disease (25–26%) was registered for Canelys 0.2%, Zytron 0.125%, and Zytron 0.15% treatment variants (Table 8 and Table 9). Mimox 0.2% grouped with Zeolite 0.5% (2018 result) due to similar disease intensity. Rust disease was controlled in a similar manner through annual treatment application of Mimox 0.3% and Altosan with its two concentrations for 2019.

**Figure 4:**
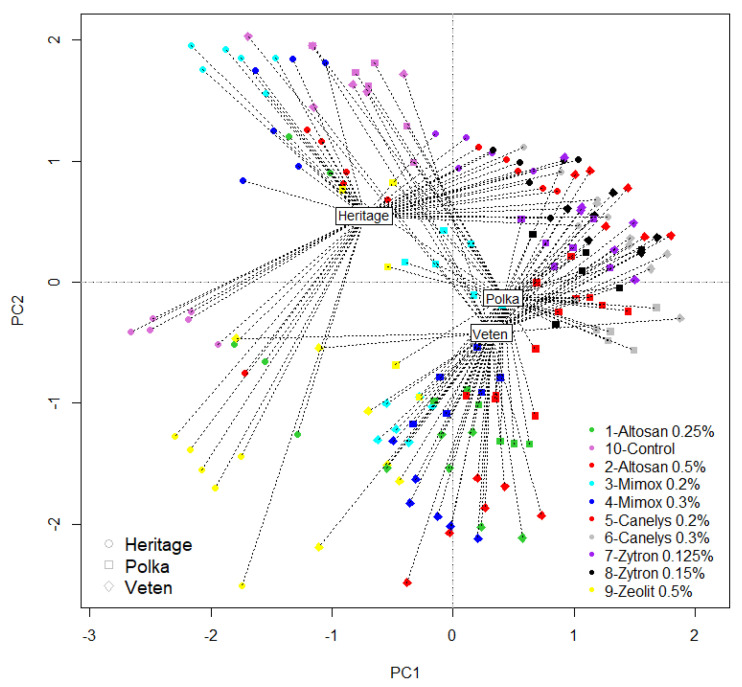
PCA raspberry rust disease treatment response. Variants tested: V1-Altosan 0.25%; V2-Altosan 0.5%; V3-Mimox 0.2%; V4-Mimox 0.3%; V5-Canelys 0.2%; V6-Canelys 0.3%; V7-Zytron 0.125%; V8-Zytron 0.15%; 9-Zeolite 0.5%; V10-Control. A full description of treatment recipes is provided in Table 12.

Raspberry experiment principal component analysis (Figure 4) concentrated most of the variants on the positive side of PC1 axis (97.07% of variants showed reduced rust disease presence). Fewer variants had shown a negative response, being situated on the PC2 plot (2.64% of treatments have not influenced rust disease severity for raspberry). Zeolite 0.5%, Mimox 0.2%, and Altosan (0.25% and 0.5%) were on the negative side. ‘Heritage’ raspberry variety was the most varied in response and had an important plot dispersion as shown in Figure 4. Moreover, it had the most evident differentiated distances between points suggesting instability. By contrast, ‘Heritage’ had the most stable but negative graphical representation of Zeolite 0.5% and Altosan 0.25% treatments. ‘Polka’ and ‘Veten’ varieties were very close on the graph as their disease occurrence was found to be quite similar and disease treatment response was also similar for Canelys 0.2% and Zytron 0.15% treatment variants. On the other hand, Mimox 0.2% had opposite reactions for these two varieties, its dispersion for ‘Polka’ variety being on the positive side. Altosan 0.5% also had a dual role positive/negative but only offered stable response in case of the ‘Veten’ variety.

The in vivo trial results for both species overlapped the in vitro ones for some of the tested products. The comparison between the two experiments revealed an interesting behavior of the rust pathogenic fungus and the natural compounds aiming to reduce its negative effect on plants. Altosan 0.5% was the only product that had shown a better field response to rust disease control than the outcomes obtained in laboratory testing. Zeolite 0.5% confirmed nearly half of its lab efficiency. Surprisingly, Mimox 0.3% had the largest range for efficiency out of all tested natural-based products.

In comparison with the blackberry global results, raspberry field reactions to the two experimental years were grouped in more numerous, but smaller, clusters with less predictability patterns. Raspberry varieties responded differently for every year tested and fewer products presented stability. Rust disease prevalence was not as high for raspberry as for blackberry plants. The variety resistance to disease and the annual climate characteristics might have altered the response for some varieties (e.g., ‘Polka’ and ‘Veten’). Raspberry rust disease attack ranged from 5.35–33.25% for Canelys 0.3% and untreated witness. Although blackberry plants had been more affected by rust, disease attack range was broader for this species (7.18–56.71%), the same products had higher rate efficiency in this case, for the studied pathogen, than in raspberry. Blackberry varieties reactions were more stable in time for many of the products tested, rather than raspberry’s reactions to the applied treatments. This outlined only few products for predictability traits for this species.

Cluster and PCA analysis showed stability traits of the tested variants as well as uniformity and data dispersion for each variant investigated. All six varieties confirmed that the most significant efficiency over *P. rubi-idaei* and one of the most stable treatment variants was Canelys 0.3%. Cinnamon is known for its fungistatic activity and the present study confirmed its high performance with a 70–75% efficiency on rust disease control for both species analyzed. Furthermore, a predictable outcome, although not efficient enough for rust organic control management was generated by Zeolite 0.5%. Zeolite’s low performance, close to the control, may be determined either by the concentration applied or by its capacity to retain humidity and thus, affect the plants evapotranspiration and photosynthesis capacity because it creates a white film on all sprayed surfaces. This phenomenon was also observed during the fruiting season for both species investigated and led to acceleration of fruit ripening. The impact on fruit ripening of Zeolite could be documented by further studies. Mimox 0.3% was also a stable variant for both species and managed to offer an average efficiency of 30% for raspberry and of 15–20% for blackberry rust disease control. Although previous studies showed a better inhibitory capacity on fungi for *Mimosa* plant extract, the present study did not confirm this. This may be the result of a specific reaction of these fungi to the natural compound of the product or a certain species rust relation over time.

Zytron in its two tested concentrations (0.125% and 0.15%) tended to show a response pattern for some of the varieties investigated and did not show prominent stability. Although disease attack values were quite tight between tested variants of the same product, the higher concentration had a better efficiency against rust disease, as expected. The citrus-extract-based product managed to reduce rust by 50% for blackberry and 60–70% for raspberry, respectively. Altosan was one of the products that had a large difference as far as efficiency was concerned between species and was also less stable for future response predictability. For the blackberry varieties analyzed it managed to control the pathogen by roughly 20%, whereas for raspberry its efficiency doubled (40%).

## 3. Discussion

This study is one of the few concerning organic control of rust disease. According to our knowledge, there are no recent scientific reports on raspberry and blackberry rust pathogen management. Thus, the results of this study provide important information in this respect.

In the present study we tested different natural-based products with fungistatic properties, although they were not specifically formulated for *P*. *rubi-idaei* control. Furthermore, no previous studies were made to determine the role of all tested products in mycelia growth of the rust fungi. Canelys and Mimox/Mimoten were studied for their insecticidal properties on *Artemia franciscana* ”brine shrimp” [33]. In a study by Kazlauskaitė et al. [34] the product Canelys positively influenced the growth of sweet basil. Zytron and Mimox are among other products tested for fungistatic value on *Alternaria solani* fungus by Mălinaș et al. [35] while Altosan and Zeolite have no scientific reports on their properties previously mentioned in literature.

Recent studies have found that essential oils and natural plant extracts are very effective against fungal pathogens and showed high inhibition levels of conidia germination and mycelia growth [8,9,20]. Moreover, in a large study performed by Clerck et al. [36] 90 EO’s were tested in vitro for their antifungal effects against 10 plant pathogens and most of them proved an efficacy comprised between 67 and 100% against most common phytopathogens like *Fusarium* spp. and *Botrytis cinerea. Aspergillus* spp. are also common pathogens for which plant extracts and EO’s have proven fungistatic effects. Thyme and oregano had shown a high impact, whereas *Aspergillus* spp. were found to be less susceptible to cinnamon [5]. By contrast, cinnamon methanol extract can significantly inhibit *A. niger* growth and also the growth in *Botrytis cinerea* and *Fusarium moniliforme* [15,19]. Thus, the form in which a natural compound is stabilized as a fungistatic product is important and can generate different outcomes for the inhibitory growth of the same mycelia [37,38].

In a study conducted by Hu et al. [39], cinnamon EO showed 93.51% mycelial growth inhibition against *Alternaria* species. Moreover, Citrus EO showed 86.66% fungal mycelial growth inhibition for *Aspergillus flavus*, 70.66% for *A. terreus* and 65.66% for *Fusarium culmorum*, a very common wheat pathogen and 100% inhibition at 50 µL/mL. Further studies will determine the optimal concentration to prevent tissue burn [39]. *Citrus lemon* ethanolic leaf extracts showed 61% inhibition rate for *Alternaria* sp. in a recent study [21]. In addition, chitosan nanoparticles coatings controlled successfully mycelial growth for *Alternaria solani* (72%) and *Fusarium oxysporum* (87%) in a study by Sathiyabama and Parthasarathy [29] therefore revealing the in vitro antifungal chitosan efficacy against these phytopathogenic fungi. The *Mimosa* plant extract previously described, during in vitro testing, showed a strong antimicrobial activity against human pathogens [17,21]. Llorens et al. [26] discovered a novel function of this plant extract: enhancing the immune response to *Sclerotinia* plant pathogen. Thus, in vitro direct toxicity against *Sclerotinia* could not be achieved but it was suggested that the treatment worked by inducing the plant defense systems as a result of field observations.

Based on our results, the product with the highest inhibitory capacity on rust conidia germination was Canelys. In its formulation the basic substance is the cinnamon extract. During the first 24 h the product efficiency was 100%, and by the end of the experiment it proved a total efficiency of 84.53% at the 0.3% concentration and of 80.42% for Canelys 0.2% (values were calculated by reference to total germination recorded after 48 h for the control group using the mathematical method: simple rule of three). Previous studies conducted by Ćosić et al. [7] on the antifungal capacity of the cinnamon showed high inhibition values, over 90%, or even 100% for certain pathogens according to the results obtained by Hu et al. [39]. The present study confirmed the high level of effectiveness of the tested product, providing equally important values although not as high as the ones stated above. Furthermore, significant values were reported for the product based on citrus extract, Zytron, which had the best effect at its higher concentration tested (78.34%), confirming the results previously found in the literature [22,40]. In particular, in a recent study on citrus EO antifungal activity, Elgatet al. [19] found higher fungistatic values (86.66–100%) against different molds.

The increased bactericide and fungistatic capacity of the Chitosan was debated in extensive studies conducted by Goy et al. [15] and Ma et al. [41]. The product Altosan, which has in its formulation the marine polymers of Chitosan, did not confirm a capacity to inhibit the germination of rust conidia as high as the previous indications found in literature. Thus, in vitro experiments results showed a degree of inhibition of 31.95% for the Altosan 0.5% variant. Although the Chitosan treated variant did not show an important fungistatic reaction in the present research, a recent study by Tan et al. [16] on novel cationic chitosan derivate functionalized with triphenylphosphonium salt revealed over 80% inhibitory effect against *Botrytis cinerea* at 1.0 mg/mL. Outcomes from this study may suggest a better formulation for chitosan-based products like Altosan. Moreover, the results found for chitosan (Altosan) contradict previous studies regarding antifungal strong inhibitory capacity [14,15,29].

The smallest value of germination inhibition percentage reported was by Zeolite (20.6%), although at the first interval of 24 h after the sowing of the rust spores the volcanic tuff showed a high level of inhibition of germination. A very recent study led by Cui et al. [32] attests the high inhibitory capacity of zeolite-exchanged ions, up to 99%, on resistant human pathogens such as *Staphylococcus aureus* and *Candida albicans*. The incorporation of Ag cations in zeolite particles was documented previously for fungicidal and antimicrobial effects [42,43].

The results of the field assays performed in this study largely confirmed those of the investigations done in laboratory regarding the effects of pre-tested natural-based substances on rust. Thus, the highest values of the means, according to the ANOVA test, showed the smallest effects in controlling *P*. *rubi*-*idaei* in organic farming for the following natural-based products: Zeolite, Mimox and Altosan. This applies vice versa for the small values of the means. Canelys and Zytron natural-based products have been proven to possess the best effectiveness in rust control. The uniformity of the data in the tested variants showed small differences between the two concentrations applied for each product except for Zeolite. However, in the case of Mimox and Altosan products the most pertinent results were obtained in the variants treated with higher concentrations.

The two tested berry species, and the six varieties observed, showed different reactions to the application of treatments. The results collected by performing data analysis through the LSD test revealed no significant differences in the batch cultivated with ‘Chester’ and ‘Loch Ness’ blackberry varieties, after treatment spraying with Altosan 0.25% and Mimox 0.3% natural-based products. Furthermore, these varieties had less statistical differences for other tested variants rather than ‘Thorn Free’. By contrast, the raspberry cultivated field with ‘Heritage’, ‘Veten’, and ‘Polka’ varieties reported that Mimox 0.3%, Canelys 0.2%, and Altosan 0.25% variants had significant differences. These results indicate that the variety itself plays a role in the resistance towards the plant pathogen.

Further studies on rust inherited infection and treatment response could provide a more comprehensive approach on organic disease management for this fungus. Moreover, field trials applying other concentrations for the natural-based substances investigated in this study as well as the analysis of the synergistic effect of combining the active elements contained in the products (for two or more products in a single application), could provide further and interesting information on the most effective control strategy for *P. rubi-idaei* in organic farming.

## 4. Materials and Methods

### 4.1. Sampling Material and Natural-Based Products Origin and Use

The present study was carried out over a period of two years (2018–2019). During this time, the attack of *P. rubi-idaei* (DC) P. Karst. on the leaves and canes of berry plants was assessed by observations performed 6 times/year. In particular, the presence of rust spores was checked through microscopical analyses done for 6 plant varieties belonging to two species: blackberry (‘Chester’, ‘Thorn Free’, ‘Loch Ness’) and raspberry (‘Heritage’, ‘Veten’, ‘Polka’).

Microscopical observations of leaves and sprouts revealed a massive presence of spores of *P*. *rubi*-*idaei* on the investigated varieties. A total of 36 samples (leaves) infected by this pathogen have been collected also for in vitro testing of 5 antifungal products recommended in organic farming. The tested products, based on natural extracts, were: Altosan (AltincoAgro, Spain), Mimox/Mimoten (AltincoAgro, Spain), Canelys (Atlántica Agricola S.A., Spain), Zytron (Atlántica Agricola S.A., Spain), and Zeolite (Zeolites Production S.A., Romania). Details about the natural products-based treatments (including their active substance) which were applied to the above-described blackberry and raspberry varieties are provided in Table 12.

### 4.2. In Vitro Experiments

In order to determine the influence of organic products on the germination of rust spores, 12 samples have been prepared. Samples of two Petri dishes were assigned to each tested substance and the reporting was made to the untreated witness. Each treatment has been replicated three times. Before the samples were prepared, the vegetative organs collected from the field were previously chilled at 5 °C temperature. The rust spores were removed with a sterilized stainless-steel spatula and applied through pipette dispersion, under sterile conditions, on PDA culture media (Ingen Laboratory, Timișoara, Romania) in Petri dishes (Ø 90 mm). The rust inoculum was of 100 CFU for each Petri and was obtained from a four time dilution of a 1 × 10^6^ spore/mL suspension. Subsequently, the Petri dishes were incubated for 48 h at 20 °C, protected from light with an opaque filter sheet. Only samples that had a germination of at least 30% compared to the control were declared valid for the experiment according to recommendations from previous studies by Bruzzese and Hasan [3]. Germinated teliospores were considered the ones which doubled the initial size of the granuloma. Each sample was analyzed at intervals of 24 h and 48 h, respectively.

### 4.3. Field Testing of the Natural-Based Products

In parallel to the above-described tests performed in vitro, the plant’s reaction after the application of the five earlier mentioned natural-based products in the field was also tested for two years in a row. Frequency of the disease, intensity of the infection and the degree of rust attack for each treated batch were calculated in order to observe the differences between the untreated and treated blackberry and raspberry varieties.

The in vivo study was conducted in an experimental field located in the Transylvanian Plain, in the locality of Grebenişu de Câmpie, Mureş County, Romania (46°36′ N and 24°17′ E). Each plant had 4.5 m^2^ nutrition space. No fertilizers were applied in the field, in order to examine the response of plants to eco-pedological conditions. Soil was a cambisoil type, with a medium nutrient assurance.

Six symmetrical batches were used, cultivated with organic blackberry varieties ‘Chester’, ‘Thorn Free’, ‘Loch Ness’ and organic raspberry ‘Heritage’, ‘Veten’, and ‘Polka’ varieties, respectively. The organic products tested were: Altosan, Mimox, Canelys, Zytron, and Zeolite. A total number of 9 variants of treatments were applied, each with 3 replicates. For each batch one has been left untreated to serve as control. In both years investigated, 2018 and 2019, the same batches were treated with the same product concentrations for each variety as reported in Table 12.

The distance between plants was 1.5 m at a time within the rows and 3 m between the rows. A buffer space of 3.5 m was left between the two batches. Variants with the same number of plants were prepared at a time. The application of the treatments was done as prevention from the swelling of the buds, corresponding to BBCH 6 until the harvesting of the fruits, (BBCH 8) according to Schmidt et al. (2001), and a total of six treatments were applied for the whole period. During the spraying application of the treatments, a plastic panel was used to limit contamination to other variants. The reapplication of the treatments was repeated every 10 days.

In order to certify the presence of the disease in experimental batches, 10 plants per batch were randomly selected and analyzed by weekly observations during rust attack. The processed data came from the observations registered in the period 27 June–30 July (2018), respectively 22 June–26 July (2019) when the presence of rust conidia on the vegetal organs was high (BBCH 8) [44]. These observations were centralized following the application of the three repetitions related to each variant. Subsequently, the Frequency (F), Intensity (I) and the Degree of Attack (DA) were calculated (www.istis.ro, accesed on 16 February 2021). All these parameters were estimated for each variant using well known formulas as described in Table 13.

### 4.4. Data Analysis of Results

All the data were analyzed with R Studio software version 1.4.1106 [45], on R platform [46], with basic statistics extracted by formulas from package “psych” [47]. Prior to the assessment of the treatments impact over the evolution of *P. rubi-idaei*, all data were subjected to the two-way ANOVA test performed in the “agricolae” package [48]. The same package was used for the post-hoc exploration of the present research results with the least significant difference (LSD) test at *p* < 0.05 [49]. For a complete analysis, the LSD test was applied for each species, in order to assess the inter-annual differences between varieties [50]. To further explore the similarities within the combination of variety *x* treatment, a cluster analysis was performed with the “ape” package [51]. The final step was represented by the ordination applied to all observations, which offered a visual form of variety response similarity to the applied treatments, and the differences between varieties induced by treatments. Principal component analysis (PCA) was also performed with “vegan” package [52].

## 5. Conclusions

From the analysis of all substances tested in this study, both in vitro and in vivo, it can be concluded that the product based on cinnamon extract (Canelys), and the citrus-based (Zytron) product were the most effective in controlling the rust pathogen in berry plantations. Moreover, all concentrations analyzed for Canelys and Zytron showed an above-average efficiency during in field tests in all six varieties tested as far as the attack degree was concerned.

Zeolite failed to confirm its potential (20.6% germination inhibition) under field conditions expressing frequency and intensity close to the witness. Based on these results, it can be concluded that it should not be recommended for the control of rust disease in organic blackberry and raspberry plantations. Moreover, Altosan (containing chitosan poliglucosamine) at both concentrations analyzed, had a higher fungistatic action in field, rather than under lab conditions. Further studies on the product performance under specific weather conditions may offer a better future approach on the mater.

It was observed that Mimox (based on *Mimosa* bark extract) performance was influenced by the species sensibility towards rust pathogen. For example, Mimox treated blackberry plants expressed higher infection rates (‘Loch Ness’ 69% F and 72.45% I) than raspberry ones (‘Polka’ 35.67% F and 41.13% I). Furthermore, blackberry species had a weaker response to rust control products application, than raspberry species for the whole study.

Outcomes from the Zeolite use allowed to conclude that it is not recommended for rust control in organic raspberry and blackberry plantations at tested concentration (0.5%). Furthermore, its white residues that present on fruits and leaves can cause serious commercial depreciation.

Above all, results of the present investigation could offer an important scientific contribution to the disease control management of rust for *Rubus* spp. and help in filling in the gap which still exists in literature. Further long-term research on *P*. *rubi*-*idaei* reaction to the products tested for both species, raspberry and blackberry, will lead to an indicative program to control this pathogen in organic berry farms.

In addition, the present research undertaken contributes to a coherent plant disease management for organic farms of scientifically proved efficiency for fungistatic products which can be a great tool for farmers that grow organic raspberries and blackberries.

## Figures and Tables

**Table 1:** Influence of in vitro tested substances on rust conidia germination at different time intervals.

Treatment	24 h	48 h
Altosan 0.25%	12.67 ± 2.08 ^b^	25.33 ± 3.06 ^b^
Altosan 0.5%	9.00 ± 4.00 ^bc^	24.00 ± 3.61 ^bc^
Mimox 0.2%	16.67 ± 3.06 ^a^	22.00 ± 4.58 ^bc^
Mimox 0.3%	8.33 ± 1.53 ^c^	18.67 ± 1.53 ^c^
Canelys 0.2%	0.00 ± 0.00 ^d^	6.33 ± 5.13 ^d^
Canelys 0.3%	0.00 ± 0.00 ^d^	5.00 ± 1.00 ^d^
Zytron 0.125%	1.67 ± 2.08 ^d^	10.33 ± 2.52 ^d^
Zytron 0.15%	0.33 ± 0.58 ^d^	7.00 ± 1.00 ^d^
Zeolite 0.5%	0.67 ± 1.15 ^d^	25.67 ± 4.73 ^b^
Control	12.00 ± 3.61 ^a^	32.33 ± 2.52 ^a^
F test	23.52 *p* < 0.001	26.12 *p* < 0.001

Note: Values presented in the table are the means ± standard error. Means followed by different letters indicate differences at *p* < 0.05 according to LSD test. Variants tested: V1-Altosan 0.25%; V2-Altosan 0.5%; V3-Mimox 0.2%; V4-Mimox 0.3%; V5-Canelys 0.2%; V6-Canelys 0.3%; V7-Zytron 0.125%; V8-Zytron 0.15%; 9-Zeolite 0.5%; V10-Control. A full description of treatment recipes is provided in Table 12.

**Table 2:** Two-year (2018–2019) ANOVA analysis of the interactions between different variables and the fungal pathogen presence (*p* < 0.001) in blackberry varieties.

Variable	Frequency(%)		Intensity(%)		Degree of Disease Attack (%)	
	*F stat*	*p.val*	*F stat*	*p.val*	*F stat*	*p.val*
Y	33.46	*p* < 0.001	32.03	*p* < 0.001	13.52	*p* < 0.001
V	2765.97	*p* < 0.001	2439.13	*p* < 0.001	2926.04	*p* < 0.001
T	325.41	*p* < 0.001	777.82	*p* < 0.001	595.62	*p* < 0.001
Y × V	24.50	*p* < 0.001	102.44	*p* < 0.001	44.30	*p* < 0.001
Y × T	1.55	0.139	2.57	*p* < 0.001	0.65	0.748
V × T	10.47	*p* < 0.001	15.74	*p* < 0.001	26.85	*p* < 0.001
Y × V × T	1.47	0.113	3.95	*p* < 0.001	1.09	0.367

Note: Y—year; V—variety; T—treatment.

**Table 3:** Variant interactions with blackberry varieties for a period of two years (2018–2019) after ANOVA analysis (*p* < 0.001).

Variety		Frequency(%)		Intensity(%)		Degree of Disease Attack (%)	
		*F stat*	*p.val*	*F stat*	*p.val*	*F stat*	*p.val*
Thorn Free	Year	99.22	*p <* 0.001	157.13	*p <* 0.001	128.45	*p <* 0.001
	Treatment	100.08	*p <* 0.001	137.79	*p <* 0.001	105.33	*p <* 0.001
Chester	Year	0.90	0.348	3.71	0.061	0.58	0.451
	Treatment	135.43	*p <* 0.001	438.06	*p <* 0.001	287.29	*p <* 0.001
Loch Ness	Year	0.00	0.948	14.52	*p <* 0.001	5.57	0.023
	Treatment	101.98	*p <* 0.001	337.88	*p <* 0.001	222.00	*p <* 0.001

**Table 4:** ‘Chester’ blackberry variety response to rust disease treatments applied during 2018–2019.

Treatment	Frequency (%)	Intensity (%)	Degree of Disease Attack (%)
Altosan 0.25%	63.17 ± 0.70 ^de^	67.92 ± 0.73 ^de^	42.90 ± 0.81 ^hi^
Altosan 0.5%	60.33 ± 0.71 ^e^	65.70 ± 0.86 ^e^	39.63 ± 0.75 ^j^
Mimox 0.2%	62.00 ± 0.93 ^de^	71.35 ± 0.93 ^bcd^	44.23 ± 0.75 ^gh^
Mimox 0.3%	60.67 ± 0.80 ^e^	66.82 ± 0.90 ^e^	40.57 ± 0.96 ^Ij^
Canelys 0.2%	41.00 ± 0.97 ^lm^	39.93 ± 0.39 ^lm^	16.40 ± 0.51 ^qr^
Canelys 0.3%	39.17 ± 0.79 ^m^	38.85 ± 0.67 ^m^	15.27 ± 0.56 ^r^
Zytron 0.125%	49.17 ± 1.14 ^gh^	42.77 ± 0.72 ^jkl^	21.07 ± 0.65 ^op^
Zytron 0.15%	46.67 ± 0.95 ^hij^	44.22 ± 0.64 ^hijk^	20.60 ± 0.64 ^op^
Zeolite 0.5%	69.17 ± 0.70 ^bc^	68.88 ± 0.83 ^cde^	46.97 ± 0.77 ^efg^
Control	67.83 ± 0.95 ^c^	71.07 ± 0.68 ^bcd^	48.25 ± 1.10 ^de^

Note: Values presented in the table are the means ± standard error. Means followed by different letters indicate differences at *p* < 0.05 according to LSD test applied to all varieties from each species. Treatment variants: V1-Altosan 0.25%; V2-Altosan 0.5%; V3-Mimox 0.2%; V4-Mimox 0.3%; V5-Canelys 0.2%; V6-Canelys 0.3%; V7-Zytron 0.125%; V8-Zytron 0.15%; 9-Zeolite 0.5%; V10-Control. A full description of treatment recipes is provided in Table 12.

**Table 5:** ‘Thorn Free’ blackberry variety response to rust disease treatments applied during 2018–2019.

Treatment	Frequency (%)	Intensity (%)	Degree of Disease Attack (%)
Altosan 0.25%	42.83 ± 0.79 ^kl^	47.43 ± 2.70 ^gh^	20.38 ± 1.42 ^op^
Altosan 0.5%	40.83 ± 0.54 ^lm^	45.8 ± 3.25 ^hij^	18.78 ± 1.54 ^pq^
Mimox 0.2%	45.5 ± 0.99 ^ijk^	47.2 ± 1.81 ^ghi^	21.55 ± 1.28 ^nop^
Mimox 0.3%	41.33 ± 0.76 ^lm^	46.68 ± 2.01 ^ghi^	19.33 ± 1.08 ^p^
Canelys 0.2%	31 ± 1.81 ^n^	29.78 ± 1.40 ^n^	9.35 ± 0.98 ^s^
Canelys 0.3%	28.5 ± 2.01 ^n^	25.08 ± 0.96 ^p^	7.18 ± 0.73 ^s^
Zytron 0.125%	30.83 ± 2.09 ^n^	29.42 ± 1.65 ^no^	9.75 ± 1.31 ^s^
Zytron 0.15%	29 ± 1.88 ^n^	25.73 ± 0.55 ^op^	7.52 ± 0.63 ^s^
Zeolite 0.5%	44.83 ± 0.54 ^jk^	49.65 ± 2.54 ^g^	22.5 ± 1.41 ^mno^
Control	48.33 ± 1.82 ^hi^	50.37 ± 1.76 ^g^	24.5 ± 1.76 ^m^

Note: Values presented in the table are the means± standard error. Means followed by different letters indicate differences at *p* < 0.05 according to LSD test applied to all varieties from each species. Treatment variants: V1-Altosan 0.25%; V2-Altosan 0.5%; V3-Mimox 0.2%; V4-Mimox 0.3%; V5-Canelys 0.2%; V6-Canelys 0.3%; V7-Zytron 0.125%; V8-Zytron 0.15%; 9-Zeolite 0.5%; V10-Control. A full description of treatment recipes is provided in Table 12.

**Table 6:** Significance of applied treatments for *Phragmidium rubi-idaei* control in ‘Loch Ness’ blackberry variety during 2018–2019.

Treatment	Frequency (%)	Intensity (%)	Degree of Disease Attack (%)
Altosan 0.25%	70.17 ± 0.31 ^bc^	71.17 ± 0.31 ^bcd^	49.97 ± 0.41 ^cd^
Altosan 0.5%	68.17 ± 0.31 ^c^	66.53 ± 0.39 ^e^	45.37 ± 0.44 ^fgh^
Mimox 0.2%	71.67 ± 0.67 ^b^	72.45 ± 1.45 ^abc^	51.93 ± 1.23 ^fc^
Mimox 0.3%	69.00 ± 0.93 ^bc^	69.27 ± 0.71 ^bcde^	47.83 ± 1.10 ^def^
Canelys 0.2%	55.17 ± 0.48 ^f^	43.67 ± 0.43 ^ijk^	24.08 ± 0.34 ^mn^
Canelys 0.3%	51.33 ± 0.76 ^g^	41.63 ± 0.47 ^klm^	21.40 ± 0.53 ^nop^
Zytron 0.125%	64.17 ± 0.60 ^d^	55.58 ± 1.03 ^f^	35.70 ± 0.95 ^k^
Zytron 0.15%	60.50 ± 0.92 ^e^	54.38 ± 0.74 ^f^	32.88 ± 0.87 ^l^
Zeolite 0.5%	71.33 ± 1.05 ^b^	75.75 ± 0.75 ^a^	53.43 ± 0.92 ^b^
Control	78.00 ± 0.89 ^a^	72.70 ± 1.08 ^ab^	56.73 ± 1.31 ^a^

Note: Values presented in the table are the means ± standard error. Means followed by different letters indicate differences at *p* < 0.05 according to LSD test applied to all varieties from each species. Treatment variants: V1-Altosan 0.25%; V2-Altosan 0.5%; V3-Mimox 0.2%; V4-Mimox 0.3%; V5-Canelys 0.2%; V6-Canelys 0.3%; V7-Zytron 0.125%; V8-Zytron 0.15%; 9-Zeolite 0.5%; V10-Control. A full description of treatment recipes is provided in Table 12.

**Table 7:** Correlations between the tested rust disease control products and three raspberry varieties for a two-year period of investigation. ANOVA test for differences (*p* < 0.001).

Variable	Frequency (%)		Intensity (%)		Degree of Disease Attack (%)	
	*F stat*	*p.val*	*F stat*	*p.val*	*F stat*	*p.val*
Y	44.88	0.000	42.04	0.000	39.39	0.000
V	346.64	0.000	249.33	0.000	325.87	0.000
T	167.36	0.000	242.99	0.000	186.63	0.000
Y × V	1.94	0.148	0.03	0.973	0.06	0.938
Y × T	0.49	0.879	0.73	0.682	0.65	0.754
V × T	7.38	0.000	8.77	0.000	10.03	0.000
Y × V × T	0.32	0.996	0.39	0.988	0.27	0.999

Note: Y—year (2018–2019); V—variety; T—treatment.

**Table 8:** Annual interaction between raspberry varieties and applied treatments against rust disease. ANOVA test for differences (*p* < 0.001).

Variety		Frequency (%)		Intensity (%)		Degree of Disease Attack (%)	
		*F stat*	*p.val*	*F stat*	*p.val*	*F stat*	*p.val*
Heritage	Year	5.33	0.026	15.73	0.000	9.55	0.004
	Treatment	86.91	0.000	137.40	0.000	104.28	0.000
Polka	Year	30.23	0.000	17.22	0.000	23.17	0.000
	Treatment	52.35	0.000	44.43	0.000	48.52	0.000
Veten	Year	16.15	0.000	10.49	0.002	11.922	0.001
	Treatment	45.86	0.000	75.68	0.000	44.969	0.000

**Table 9:** Treatment influence on rust disease frequency in three raspberry varieties during 2018–2019.

Treatment	Heritage	Disease Frequency (%) Polka	Veten
Altosan 0.25%	45.33 ± 1.02 ^cd^	31.17 ± 1.08 ^ijkl^	32.17 ± 1.40 ^ij^
Altosan 0.5%	44.83 ± 0.95 ^cd^	30 ± 0.82 ^jkl^	30.33 ± 1.12 ^jkl^
Mimox 0.2%	52.33 ± 0.84 ^a^	35.67 ± 1.02 ^gh^	36.67 ± 0.49 ^fg^
Mimox 0.3%	48.83 ± 0.65 ^b^	33.5 ± 0.76 ^hi^	33.67 ± 1.02 ^hi^
Canelys 0.2%	32 ± 1.18 ^ij^	26.5 ± 0.99 ^mno^	25.33 ± 1.23 ^op^
Canelys 0.3%	28.83 ± 1.01 ^klm^	23.83 ± 0.87 ^op^	22.83 ± 1.14 ^p^
Zytron 0.125%	35.83 ± 0.98 ^gh^	28.5 ± 0.99 ^lmn^	26.33 ± 1.12 ^mno^
Zytron 0.15%	31.5 ± 0.92 ^ijk^	26.67 ± 1.17 ^mno^	25.67 ± 1.09 ^nop^
Zeolite 0.5%	47.33 ± 0.88 ^bc^	39 ± 1.37 ^ef^	40.5 ± 1.80 ^e^
Control	51.83 ± 0.75 ^a^	43.67 ± 1.17 ^d^	46.17 ± 1.30 ^bcd^

Note: Values presented in the table are the means ± standard error. Means followed by different letters indicate differences at *p* < 0.05 according to LSD test applied to all varieties from each species. Variants tested: V1-Altosan 0.25%; V2-Altosan 0.5%; V3-Mimox 0.2%; V4-Mimox 0.3%; V5-Canelys 0.2%; V6-Canelys 0.3%; V7-Zytron 0.125%; V8-Zytron 0.15%; 9-Zeolite 0.5%; V10-Control. A full description of treatment recipes is provided in Table 12.

**Table 10:** Treatment influence on rust disease intensity in three raspberry varieties during 2018–2019.

Treatment	Heritage	Disease Intensity (%) Polka	Veten
Altosan 0.25%	53.33 ± 1.96 ^bc^	39.15 ± 1.11 ^gh^	42.35 ± 1.47 ^fg^
Altosan 0.5%	50.02 ± 1.95 ^cd^	36.23 ± 1.37 ^h^	41.25 ± 1.78 ^g^
Mimox 0.2%	55.72 ± 1.06 ^b^	39.95 ± 1.13 ^g^	46.35 ± 0.77 ^e^
Mimox 0.3%	52.5 ± 1.16 ^bc^	41.13 ± 1.23 ^g^	45.07 ± 0.91 ^ef^
Canelys 0.2%	32.33 ± 1.16 ^i^	28.97 ± 1.08 ^ijkl^	24.7 ± 1.21 ^mn^
Canelys 0.3%	28.77 ± 0.96 ^jkl^	26.75 ± 1.04 ^klm^	23.18 ± 0.95 ^n^
Zytron 0.125%	36.38 ± 1.14 ^h^	29.93 ± 1.15 ^ijk^	26.47 ± 0.93 ^klmn^
Zytron 0.15%	31.42 ± 1.10 ^ij^	28.47 ± 1.49 ^jkl^	25.73 ± 1.20 ^lmn^
Zeolite 0.5%	63.03 ± 0.73 ^a^	45.48 ± 0.73 ^ef^	51.95 ± 1.84 ^c^
Control	64.1 ± 1.04 ^a^	44.78 ± 1.05 ^ef^	47.65 ± 1.66 ^de^

Note: Values presented in the table are the means ± standard error. Means followed by different letters indicate differences at *p* < 0.05 according to LSD test applied to all varieties from each species. Variants tested: V1-Altosan 0.25%; V2-Altosan 0.5%; V3-Mimox 0.2%; V4-Mimox 0.3%; V5-Canelys 0.2%; V6-Canelys 0.3%; V7-Zytron 0.125%; V8-Zytron 0.15%; 9-Zeolite 0.5%; V10-Control. A full description of treatment recipes is provided in Table 12.

**Table 11:** Organic control of rust disease severity in three raspberry varieties during 2018–2019.

Treatment	Heritage	Degree of Disease Attack (%) Polka	Veten
Altosan 0.25%	24.25 ± 1.28 ^cd^	12.27 ± 0.77 ^klmn^	13.72 ± 1.06 ^jk^
Altosan 0.5%	22.52 ± 1.28 ^de^	10.92 ± 0.69 ^lmno^	12.62 ± 0.99 ^klm^
Mimox 0.2%	29.23 ± 1.01 ^b^	14.3 ± 0.80 ^jk^	17.00 ± 0.50 ^hi^
Mimox 0.3%	25.67 ± 0.86 ^c^	13.82 ± 0.72 ^jk^	15.23 ± 0.75 ^ij^
Canelys 0.2%	10.4 ± 0.76 ^mnop^	7.72 ± 0.57 ^qrs^	6.32 ± 0.60 ^rs^
Canelys 0.3%	8.33 ± 0.58 ^pqr^	6.42 ± 0.49 ^rs^	5.35 ± 0.48 ^s^
Zytron 0.125%	13.1 ± 0.76 ^jkl^	8.58 ± 0.63 ^opqr^	7.03 ± 0.53 ^rs^
Zytron 0.15%	9.95 ± 0.63 ^nopq^	7.67 ± 0.72 ^qrs^	6.65 ± 0.57 ^rs^
Zeolite 0.5%	29.85 ± 0.88 ^b^	17.77 ± 0.86 ^gh^	21.20 ± 1.69 ^ef^
Control	33.25 ± 1.02 ^a^	19.62 ± 1.00 ^fg^	22.57 ± 1.76 ^de^

Note: Values presented in the table are the means± standard error. Means followed by different letters indicate differences at *p* < 0.05 according to LSD test applied to all varieties from each species. Variants tested: V1-Altosan 0.25%; V2-Altosan 0.5%; V3-Mimox 0.2%; V4-Mimox 0.3%; V5-Canelys 0.2%; V6-Canelys 0.3%; V7-Zytron 0.125%; V8-Zytron 0.15%; 9-Zeolite 0.5%; V10-Control. A full description of treatment recipes is provided in Table 12.

**Table 12:** Natural products-based treatments applied to the blackberry and raspberry varieties investigated in this study during 2018–2019.

Treatment/Product	Concentration (%)	Active Substance
V1/Altosan	0.25%	Chitosanpoliglucosamine (4%)
V2/Altosan	0.5%	
V3/Mimox	0.2%	*Mimosa* Bark extract (80%)
V4/Mimox	0.3%	
V5/Canelys	0.2%	Cinnamon extract (70%)
V6/Canelys	0.3%	
V7/Zytron	0.125%	Citrus seed extract (20%)
V8/Zytron	0.15%	
V9/Zeolite	0.5%	Natural zeolite tuff powder (0.5%)
V10/Control	-	-

**Table 13:** Parameters estimated in field testing trials of natural-based products treatments applied on organic blackberry and raspberry during 2018–2019.

Parameter	Calculation Formula
Frequency (F%)	F % = n × 100*/N where: n = number of disease attacked organs; N = number of observed organs; * = total number of vegetative organs observed.
Intensity (I%)	I % = Σ (i × f)/N where: i = intensity of the disease attack appreciated by a grade scale (1–6) given as follows: 1 = 1–3%, 2 = 4–10%, 3 = 11–25%, 4 = 26–50%, 5 = 51–75%, 6 = 76–100%; f = number of vegetative organs that showed the degree of attack estimated by (i); N = total number of attacked vegetative organs.
Degree of Disease Attack (DA%)	DA = F × I/100, where: F–frequency of attack and I = intensity of attack.

## Data Availability

The data presented in this study are available in the article.
